# Latent Gonorrhœa

**Published:** 1877-01

**Authors:** 


Latent Gonorrhœa, Especially with Regard to its Influence on Fertility in Women.—Dr. E. Noeggerath, of New York, read a paper before the American Gynæcological Society at its recent meeting in New York. In this paper it was maintained that gonorrhœa, when once contracted, persisted for life in certain portions of the male genital organs, notwithtanding its apparent cure. It was believed that that fact explained, to some extent at least, why uterine disease was so prevalent in our large cities; why blooming girls faded so soon after marriage; why treatment of certain cases of uterine disease so often failed; and also why sterility was so prevalent. The germs of the disease remained concealed, and where the proper conditions were presented, it burst forth in all its virulence, and was followed by the disastrous consequences to which allusions had been made.

Dr. Noeggerath's paper was criticised sharply by Drs. Engelmann, of St. Louis; Chadwick, of Boston; Trenholme, of Montreal; and Johnson of Washington.

Dr. Engelmann, however, remarked that he had noticed, at *post mortem*, evidences of salpingitis in numerous cases which to him had heretofore been unexplained, nor had he attributed much value to them, but which, perhaps, could be explained in accordance with the theory set forth in Dr. N.'s paper. That condition had been seen especially in those cases in which gonorrhœa had been present at some previous time.—*American Journal of Obstetrics*, October, 1876.

